# Interprofessional continuing professional development programs can foster lifelong learning in healthcare professionals: experiences from the Project ECHO model

**DOI:** 10.1186/s12909-022-03500-w

**Published:** 2022-06-06

**Authors:** Sanjeev Sockalingam, Thiyake Rajaratnam, Amanda Gambin, Sophie Soklaridis, Eva Serhal, Allison Crawford

**Affiliations:** 1 Centre for Addiction and Mental Health, 1025 Queen Street West, B1 – 2nd floor, Suite 2302, Toronto, Ontario Canada; 2 University of Toronto, Toronto, Ontario Canada

**Keywords:** Lifelong learning, Continuing professional development, Project ECHO, Mental health

## Abstract

**Background:**

The success of continuing professional development (CPD) programs that foster skills in lifelong learning (LLL) has been well established. However, healthcare professionals often report barriers such as access to CPD and cost which limit uptake. Further research is required to assess how accessible CPD programs, such as those delivered virtually, impact orientation to LLL. Project Extension for Community Healthcare Outcomes (Project ECHO®) is a CPD model that has a growing body of evidence demonstrating improvements in knowledge and skills. Central to this model is the use of a virtual platform, varied teaching approaches, the promotion of multi-directional learning and provider support through a community of practice. This study aimed to explore whether participation in a provincial mental health ECHO program had an effect on interprofessional healthcare providers’ orientation to LLL.

**Methods:**

Using a pre-post design, orientation to LLL was measured using the Jefferson Scale of Lifelong Learning. Eligible participants were healthcare professionals enrolled in a cycle of ECHO Ontario Mental Health from 2017 to 2020. Participants were classified as ‘high’ or ‘low’ users using median session attendance as a cut-point.

**Results:**

The results demonstrate an increase in orientation to LLL following program participation (Pre: 44.64 ± 5.57 vs. Post: 45.94 ± 5.70, *t (*66) = − 3.023, *p* < .01, Cohen’s *d* = 0.37), with high ECHO users demonstrating greater orientation to LLL post-ECHO.

**Conclusion:**

Findings are discussed in the context of self-determination theory and suggest there may be components of CPD programs that more readily support increased motivation for LLL for interprofessional healthcare professionals.

## Background

In recent years, continuing professional development (CPD) has been positioned as a key education intervention to support healthcare professionals in initiating system improvement efforts to address gaps in quality of care [1, 2]. To achieve this goal, CPD has moved to a competency-based model where competence is not seen as static, rather competency-based CPD is contingent on healthcare providers expanding their capabilities and acquiring new knowledge and skills while in practice [3]. Healthcare professionals’ skills in lifelong learning (LLL) are critical to a competency-based model of CPD and to support healthcare professionals with keeping up-to-date with the large volume of information and advancements in healthcare [3]. As a result, graduate medical education programs and standards have identified LLL as a core competency to residency training [4]. However, existing data suggests that postgraduate training programs may not cultivate skills in LLL and may, in fact, decrease orientation to LLL over the course of medical residency training [5, 6]. As a result, CPD programs may play an important role in engaging and further developing LLL capabilities when in practice.

Although many definitions exist for lifelong learning, Hojat and colleagues have identified four components to lifelong learning: 1) self-initiated activities, 2) information-seeking skills, 3) sustained motivation to learn, and 4) the ability to self-assess and identify one’s own learning needs [7, 8]. Studies suggest that LLL is lower during medical residency and healthcare training programs and an absence of formal training in LLL in some residency programs [7]. A meta-synthesis of nurses’ perception towards CPD also found that CPD was fundamental to LLL, and to updating their skills in order to deliver safe and effective care [9]. Given the relationship between meaningful CPD and LLL, it is of interest to examine how CPD program design influences orientation to LLL.

Project Extension for Community Healthcare Outcomes (Project ECHO®) is a virtual tele-education and capacity building CPD program that aims to address gaps in specialty care for complex patients in underserved areas [10–12]. Project ECHO shares best practices through a collaborative knowledge network that relies on iterative learning loops where community-based participants (Spokes) learn from specialist team members (Hub) and other participants in a multidirectional learning model [11, 13]. Central to this CPD model is its use of multiple teaching approaches (i.e. didactic, case-based learning), and its use of an accessible (i.e. virtual and often no cost) community of practice to engage participants in the program [11, 12]. The ECHO model has growing evidence demonstrating participants’ improvements in knowledge and skills, and improved patient safety and healthcare outcomes in a range of chronic diseases [12, 14, 15]. Moreover, the ECHO model has also increased participants’ awareness and understanding of interprofessional care, including the roles of other healthcare professionals [16]. It is of interest to understand if, and how, the design of this CPD program supports LLL, especially given the increase in virtual education programs during the COVID-19 pandemic [17].

Project ECHO has been shown to have several effects on learning and capacity building for healthcare professionals and teams in clinical practice. For example, the ECHO model has been shown to foster the development of adaptive expertise, which is the application of existing knowledge to clinical situations while also generating new knowledge needed to address patients’ complex needs in specific contexts [18]. The development of adaptive expertise is central to the master adaptive learner model, an education model that involves self-regulated and continuous learning over time [19]. The master adaptive learner model consists of identifying practice or knowledge gaps, planning and setting learning goals, participating in learning, and self-assessment, all of which aligns with several aspects of LLL [19]. Therefore, if the ECHO model has been shown to foster adaptive expertise, it is possible that participation in ECHO programs may also increase healthcare professionals’ orientation to LLL.

The following study aimed to explore changes in healthcare professionals’ orientation to LLL. A pre-post design was used to determine if participation in an interprofessional mental health CPD program that leverages the Project ECHO model, namely, ECHO Ontario Mental Health (ECHO-ONMH), had an effect on participants’ orientation to LLL. The secondary aim of this study was to determine whether the level of participation, specifically the number of ECHO sessions attended, influenced orientation to LLL. We hypothesized that participation in ECHO-ONMH would be associated with higher orientation to LLL.

## Methods

### Participants and setting

ECHO-ONMH is a tele-education program offered by the Centre for Addiction and Mental Health (CAMH) and University of Toronto in Toronto, Canada. Eligible participants were healthcare professionals within the province of Ontario who were enrolled in ECHO-ONMH from 2017 to 2020. Participants were included if they attended at least one ECHO-ONMH session and completed both the pre- and post-cycle study questionnaires. Participants who did not complete both questionnaires were excluded from our analysis.

Each cycle of the ECHO-ONMH program consisted of approximately 33-weekly sessions (range 33-36 sessions/cycle) and the areas of focus for sessions consisted of common psychiatric disorders including mood disorders, anxiety, psychosis and substance use disorders. Each session included 1 or more clinical case presentation(s) by a Spoke (participant) in keeping with the Project ECHO model [10]. Each case discussion explicitly solicited interprofessional perspectives on the assessment and management of presented cases from the Spokes and Hub attendees. The Hub team consisted of: 3 psychiatrists with expertise in trauma, medical psychiatry and child psychiatry, a family physician, an addictions medicine specialist, a social worker, and a health librarian. This study protocol was reviewed and approved under Quality Projects Ethics Review (QPER reference number: 2016-17.109) by CAMH.

### Measures

#### Demographics

Demographic information, such as profession and practice setting, was captured during the ECHO-ONMH registration process. Participant attendance was recorded weekly by ECHO-ONMH operations team members as per program requirements.

#### Jefferson LLL scale

The Jefferson Scale of Lifelong Learning-Health Professions Students Version (JeffSLL-HPS) [20], an adaptation of the original Jefferson Scale of Lifelong Learning-Scale, was used to capture orientation to LLL across healthcare professionals working in primary care settings [7, 8, 21]. The Health Professions Students version of the JeffSLL was selected as other versions were physician centric and participants within ECHO were interprofessional, thus requiring a health professions version of this tool. This self-reported scale includes 14-items for which each is rated on a 4-point Likert scale (1 = Strongly Disagree to 4 = Strongly Agree); the total score is calculated by taking the sum of the item ratings (range = 14-56), with higher scores indicative of greater orientation toward LLL. The scale consists of three factors: learning beliefs and motivation, attention to learning opportunities, and skills in seeking information. The JeffSLL-HPS scale’s reliability (Cronbach alpha) has been estimated in the range of 0.77-0.86 [7, 8, 20, 22]. Comparable means on the JeffSLL across practicing physicians, pediatric residents, psychiatry residents and medical students were 46.2 [SD = 5.5], 43.0 [SD = 4.8], 43.78 [SD = 6.28], and 43.5 [SD = 4.7], respectively [6, 8, 21, 23].

### Procedure

Upon registration in the ECHO-ONMH program, participants were provided a unique identifier for completion of program evaluation surveys including pre- and post-ECHO-ONMH surveys, which included the JeffSLL-HPS. Surveys were created and administered electronically using Research Electronic Data Capture (REDCap), a secure, web-based software platform [24, 25]. Participants were provided their unique identifier and the survey links at the time of ECHO-ONMH registration and upon program completion.

### Statistical analysis

Data from participants that completed both the pre and post-ECHO ONMH surveys were included in the analysis. All data analyses were conducted using Statistical Package for the Social Sciences (SPSS) Version 25, and Microsoft Excel. Paired t-tests were used to compare JeffSLL-HPS before- and after program completion. Effect sizes for significant differences in JeffSLL-HPS score were calculated using Cohen’s d and statistical significance was defined as α = .05. Pre-post comparisons were made for overall JeffSLL-HPS scores as well as for each of the 3 JeffSLL-HPS subscales. Participant attendance was standardized and expressed as a percentage. The ECHO ONMH program had multiple enrollment periods for participants, therefore standardized attendance was calculated based on the number of sessions per cycle available given each participant’s specific enrollment date. Median ECHO-ONMH attendance for the study sample was used to divide participants into ‘highly-active’ (equal or above median) and ‘less-active’ (below median) to determine the relationship between program attendance and JeffSLL-HPS scores.

## Results

### Demographics

A total of 208 participants attended ≥1 session across the 3 ECHO-ONMH cycles included in the survey, and 67 (32%) participants completed both the pre- and post-cycle surveys. Table 1 summarizes the professional breakdown of participants that completed the pre-post surveys in comparison to the professional breakdown of the entire participant group. Similar to the composition of the interprofessional community of practice, the majority of participants who completed the pre-post surveys were social workers (37.3%), followed by nurse practitioners (20%), physicians (9%), registered nurses (3.0%), and other healthcare professionals including psychotherapists, dietitians, and pharmacists (29.8%).

**Table 1:** Distribution of survey respondents by profession and practice setting

**Profession Type**	**Full Cohort (%)**	**Study Sample (%)**
Physician	15 (7.2)	6 (9.0)
Nurse Practitioner	47 (22.6)	14 (20.9)
Registered Nurse	24 (11.5)	2 (3.0)
Social Worker/Case Worker	80 (38.5)	25 (37.3)
Other	41 (19.7)	20 (29.8)
**Total**	**208 (100)**	**67 (100)**
**Practice Setting**	**Full Cohort (%)**	**Study Sample (%)**
Primary Care	101 (48.6)	38 (56.7)
Community Mental Health	36 (17.3)	11 (16.4)
Hospital	39 (18.8)	11 (16.4)
Solo Practice	8 (3.8)	2 (3.0)
Other	24 (11.5)	5 (7.5)
**Total**	**208 (100)**	**67 (100)**

Spoke participants that completed the pre-post surveys had a mean attendance of 64 ± 24.3%, while the overall mean attendance for the program was 51 ± 29.2%.

Nearly 57% of the participants were practicing in primary care settings followed by 16.4% in hospitals, 16.4% in community mental health settings, 3% in solo practice, and 7.5% in other practice settings, a distribution which is comparable to the overall participant group (see Table 1).

### Differences in pre- and post-ECHO JeffSLL-HPS scores

**Fig. 1:**
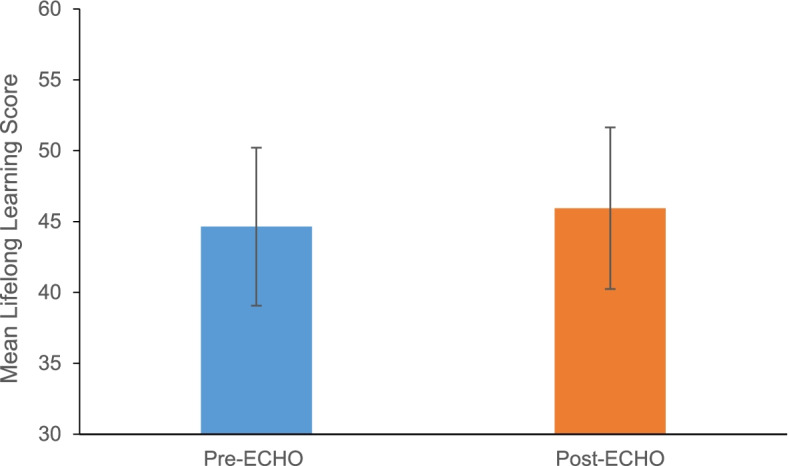
Comparison of overall LLL scores pre-post ECHO-ONMH

Analysis of JeffSLL-HPS scores showed a statistically significant increase in orientation to LLL post-ECHO (Pre: 44.64 ± 5.57 vs. Post: 45.94 ± 5.70, *t* (66) = − 3.023, *p* < 0.01, Cohen’s *d* = 0.37) (Fig. 1).

**Fig. 2:**
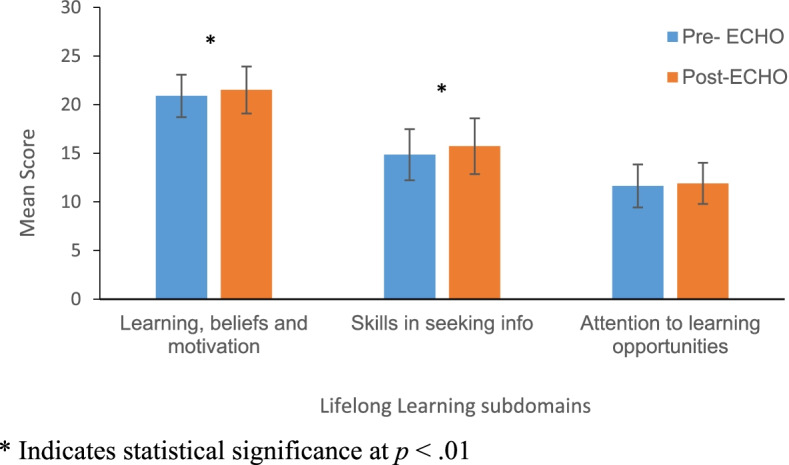
Pre-post ECHO-ONMH comparisons for each of the LLL factors. * Indicates statistical significance at *p* < .01

Analysis across each of the subdomains indicates differences in the learning beliefs and motivation subdomain (Pre-ECHO: 20.90 ± 2.18 vs. Post: 21.51 ± 2.41, *t* (66) = − 3.104, *p* < 0.01, Cohen’s *d* = 0.38), as well as the skills in seeking information domain (Pre: 14.85 ± 2.63 vs Post: 15.73 ± 2.87, *t* (66) = − 3.638, *p* = 0.001 Cohen’s *d* = 0.44. There was no significant difference in the attention to learning opportunities subdomain pre- vs. post-ECHO (Pre: 11.63 ± 2.21 vs. Post: 11.90 ± 2.12, *t* (66) = − 1.335, *p* = 0.187) (Fig. 2).

### Relationship between JeffSLL-HPS and attendance

The median session attendance of those participants who completed the surveys was 74%; a total of 31 participants attended 73% of sessions or less and were categorized as “less-active” participants. The average attendance by participants in the “less-active” group was 41.8% of sessions (range: 11-72%). A total of 36 participants attended 74% of sessions or more (“highly-active”) and the average attendance by the “highly-active” participant group was 83.4% of sessions (range: 74-97%).

**Fig. 3:**
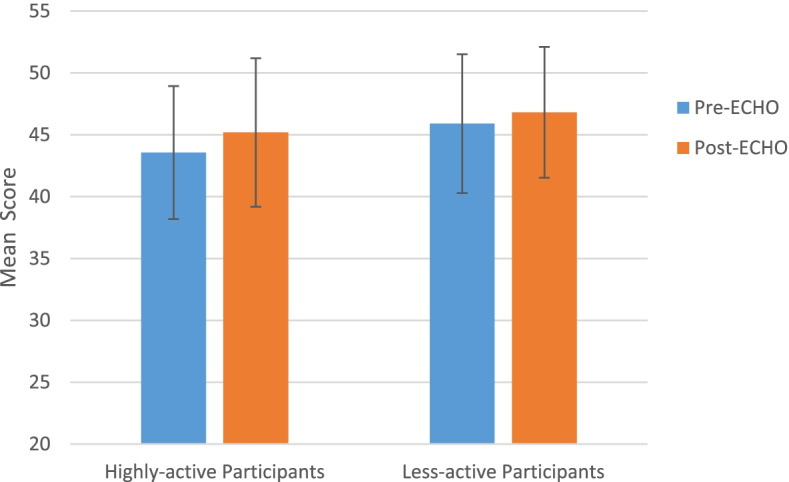
LLL scores for high and less-active ECHO participants

There was a statistically significant improvement in JeffSLL-HPS scores from pre- to post-ECHO for the participants in the “highly-active” ECHO-ONMH group (Pre: 43.56 ± 5.38 vs. Post: 45.19 ± 6.00; *t* (35) = − 3.163, *p* < .01, Cohen’s *d* = 0.52). There was no significant difference in the JeffSLL-HPS scores before vs. after ECHO-ONMH participation for the “less-active” group (Pre: 45.90 ± 5.61 vs. Post: 46.81 ± 5.29; *t* (30) = − 1.272 *p* = 0.615) (Fig. 3).

## Discussion

This study examined changes in healthcare professionals’ orientation to LLL in a provincial, interprofessional virtual CPD program, specifically ECHO-ONMH. Although a recent review has shown an increase in LLL over the course of one’s professional career (student to resident to practicing physician) [9], our sample of healthcare professionals had a mean orientation to LLL score that was lower than previous studies of practicing physicians (current study = 44.6; review of physician studies = 46.25) and rural physicians (mean = 45.56) [6, 26] To the best of our knowledge, there is no available literature on orientation to LLL for interdisciplinary CPD programs; as such, available data on physician orientation is being used as a benchmark for our findings. Despite our sample’s pre-participation orientation to LLL scores, as hypothesized, participation in this ECHO program showed a significant improvement in JeffSLL-HPS scores with an overall effect size of 0.37. Further analysis revealed significant increases in the JeffSLL domains of learning beliefs and motivation, and skills in seeking information upon program completion. No significant difference in the JeffSLL attention to learning opportunities domain was observed, which may be due to participants continuing their pre-ECHO levels of CPD engagement and participation in activities such as education rounds and conferences. Furthermore, highly active ECHO participants had significantly greater improvements in orientation to LLL, which suggests that greater exposure to ECHO sessions may be associated with greater improvement in LLL orientation.

Given the rapid implementation and expansion of virtual learning models during the COVID-19 pandemic, this study provides insights on the potential impact of virtual, interprofessional CPD programs on enhancing LLL [27] While our study did not specifically study factors influencing the association between ECHO-ONMH participation and orientation to LLL, we found a significant positive association between the change in orientation to LLL and ECHO-ONMH session attendance (i.e., highly active ECHO-ONMH participants had a significantly higher change in LLL). These findings replicate previous studies showing a positive association between participating in CPD and orientation to LLL [26].Moreover, the ECHO model utilizes a “no cost” and virtual delivery model of CPD, which aligns with previously reported factors to enhance CPD participation, specifically reduced cost and ease of access to CPD programs [28].

Self-determination theory (SDT) offers insight into how participation in this ECHO-ONMH virtual CPD program could increase learning beliefs and motivation for LLL [27]. According to Deci and Ryan, SDT aims to move individuals towards more autonomous (intrinsic) motivation by fulfilling three core needs of autonomy, relatedness and competence as they interact with the environment [29–31]. A systematic review on strategies to foster intrinsic motivation for learning identified several approaches to supporting learner autonomy, competence and relatedness that could explain how the ECHO model influences motivation for LLL [32]. First, the ECHO model utilizes multiple instructional methods (didactic, case-based discussion, opportunities for questions), supports active participation through case and content discussions, and shares educational materials and resources through a virtual community of practice; this allows participants to choose additional learning opportunities and has been associated with improved learner autonomy [32]. Learner competence can be supported by providing optimal learning challenges, structured guidance and giving constructive positive feedback [32]. Within the ECHO model, participants share case presentations and collectively participants across a range of healthcare professions “struggled” through understanding and managing patient cases with support and constructive feedback from the expert ECHO “Hub” team. Lastly, learner relatedness may have been enhanced within the ECHO model through the development of a virtual community of practice where interprofessional participants felt included, emotionally supported by both Spoke (participant) and Hub members as they learned from and with one another, and safe to have open discussions amongst peers [32]. In addition, the virtual delivery of the ECHO model further strengthens this connection and support amongst healthcare providers practicing in rural and underserved areas. Therefore, these components of the ECHO model may offer insights into how interprofessional CPD can increase motivation to learn and further increase overall LLL.

As noted in our results, ECHO-ONMH participants showed a significant improvement in information seeking skills. Although no studies have examined LLL domains in interprofessional CPD program participants, data from a physician only study suggests that physicians working in rural areas report greater challenges with information seeking skills related to LLL [26]. Our ECHO-ONMH model included a librarian (information specialist) as part of the ECHO hub (expert) team who attended each session to identify additional learning questions and shared evidence-based resources related to these clinical questions. It is possible that the identification of relevant clinical questions, review of academic and grey literature resources, and discussion of the quality of these resources increased ECHO-ONMH participants’ confidence and capabilities for information seeking. A descriptive report on the “embedded librarian” role within an ECHO Pain program further supports the notion that the rural and underserved areas may have limited access to librarians and the integration of librarians within the ECHO program may address this lack of access to evidence-based information and how to seek these resources [33]. Therefore, future research should further evaluate the impact of integrating information specialists into CPD programs on enhancing healthcare professional LLL.

The following limitations should be considered when interpreting our study results. First, although our study assessed changes in LLL after participation in a 32-36 week ECHO program, long-term data on sustained changes to LLL were not assessed in this study. Second, our data involves a sub-set of ECHO participants who consented to participate in this study and it is possible that our results may not be representative of all ECHO participants. While only 32% of ECHO-ONMH participants responded to both pre and post surveys, this response rate is within the range often reported for primary care clinicians [34]. Additionally, ECHO participants represented a range of health professions and there was variability in the level of engagement in our ECHO sample, which should support generalizability. Third, our results were based on the ECHO program model and implications for other types of interprofessional CPD programs should be examined in future studies. Additional qualitative studies are needed to provide insights into how LLL changes occur within ECHO and other virtual CPD programs.

## Conclusion

In summary, this study adds to the paucity of literature on how participation in CPD can further enhance healthcare professionals’ orientation to LLL. The results suggest that participation in a CPD programs, such as this ECHO program, may lead to increased motivation for LLL and enhance information seeking skills. The development of virtual CPD programs using specific components of the ECHO model, such as multiple instructional approaches, constructive feedback on case discussions, a safe and supportive learning environment, and connectedness through a community of practice may help enhance health professions learners’ motivation for LLL. Moreover, the involvement of additional health professions in the education and facilitation of CPD programs, such as embedded librarians, may further increase CPD learners’ LLL skills specifically related to information seeking. Future studies will need to explore changes in LLL in additional CPD programs to further elucidate factors contributing to improved LLL in CPD.

## Data Availability

The data is owned by the hospital and the ECHO Ontario Mental Health Program. The datasets used and/or analyzed during the current study are available from the corresponding author on reasonable request.

## Abbreviations

CAMH: Centre for Addiction and Mental Health
CPD: Continuing professional development
ECHO-ONMH: ECHO Ontario Mental Health
JeffSLL-HPS: Jefferson Scale of Lifelong Learning- Health Professions Students Version
LLL: Lifelong learning
Project ECHO: Project Extension for Community Healthcare Outcomes
QPER: Quality Projects Ethics Review
REDCap: Research Electronic Data Capture
SPSS: Statistical Package for the Social Sciences
